# Botho: A cultural framework for resilient human–wildlife coexistence in Botswana

**DOI:** 10.1007/s13280-026-02347-5

**Published:** 2026-03-24

**Authors:** Otshabile Bahetoleng, Amanda Stronza

**Affiliations:** Department of Ecology and Conservation Biology, Texas A&M University - Sterling C. Evans Library & Annex, 2258 TAMU, College Station, TX 77843-2258 USA

**Keywords:** Botho, Coexistence, Carnivores, Relational values, Ubuntu

## Abstract

Botho is a foundational philosophy in Sub-Saharan Africa that emphasizes interdependence, respect, and shared humanity. While it has traditionally shaped human–wildlife relationships, its present contribution to coexistence with large carnivores remains underexplored. This paper examines how Botho is a part of local decision-making in cattle farming communities of the Kalahari region of Botswana and how it shapes their choices in tolerating and coexisting with lions. Drawing on ethnographic field research, the paper describes how conservationists and cattle farmers practice Botho, and how and why it can be positive for wildlife and coexistence. The expression of Botho between social actors eases tensions, including human–human conflict over wildlife. When people treat each other with Botho, they develop empathy for one another, which sometimes extends to the wildlife. This is a potent if indirect way Botho can be a path to coexistence.

## Botho: A cultural framework for human–wildlife coexistence in Botswana

In places where humans live with wild carnivores, conservationists tend to frame human presence as a threat to carnivore survival, emphasizing risks such as persecution, habitat loss, and conflict (Woodroffe 2000). The realities and possibilities of coexistence and the cultural norms that support them are underexplored. Conflict refers to incidents of wildlife damaging crops, injuring or killing domestic animals, and threatening or killing people, or humans killing an animal or retaliating against conservation authorities (Madden 2004). By contrast, coexistence is defined as a decision humans make to share space and resources with wildlife (IUCN 2023). According to this definition, humans choose to live alongside carnivores, even though conflict is part of their reality (Hill 2021). In this way, coexistence is a continuous process of co-adaptation and reconciliation. Carnivores adjust their behaviors to avoid negative interactions with humans, and humans adjust their own behaviors to accommodate carnivores (Carter and Linnell 2023). This kind of adaptation on the human side relies on community norms, culture, ethics, and institutions that help people cope with and manage conflict. Understood this way, coexistence is not something people have. Rather, it is a dynamic relationship, between humans and wildlife, which shifts and responds to local ethics, norms, culture, and forms of governance (Carter and Linnell 2023).

Despite this understanding of coexistence, conservation research tends not to focus on choices and decisions humans are making to live with wildlife or how such decisions are upheld by local norms and culture. Instead of exploring whether people choose to share space and resources with wildlife and what the cultural underpinnings of that choice look like, researchers often assume the opposite: that coexistence is absent and must be created (Marchini et al. 2024). This assumption leads to a focus on conflict risk and strategies to "achieve coexistence," treating the definition as a blueprint for intervention rather than a lens for understanding existing relationships (Marchini et al. 2021).

When conservation researchers and practitioners treat coexistence as a fixed end state to be achieved by certain humans and wildlife, they risk missing how coexistence happens in practice. Coexistence is rarely a static goal. It is a process that unfolds in everyday life (Lute and Carter 2020). Communities often find ways to live with wildlife through small, practical choices, such as tolerating occasional livestock losses or making informal agreements about shared spaces (Jolly and Stronza 2025). These strategies are shaped by cultural norms and local values, not just by formal conservation plans (Newsom et al. 2025). Policies that recognize and support these practices can help sustain coexistence (Hartel et al. 2019; Artelle et al. 2024). Seeing coexistence this way, as dynamic, culturally informed, and context driven, rather than as a single destination, opens the door to understanding the many ways human communities all over the world are adapting to and coping with living with wildlife.

In this study, we start with the observation that cattle farmers in the Kalahari region of Botswana are choosing to live and raise livestock alongside lions, leopards, wild dogs, cheetahs, and other carnivores. We looked for evidence that the cattle farmers are choosing to live with the carnivores, to coexist and share space without intention to harm them. Outside of external laws, policies, and conservation programs, what local norms, ethics, cultural values, and decisions are shaping coexistence? We focused on the influence and relevance of the African ethic of Botho, "I am because we are.”

People's decisions about whether and how to live with wildlife may be shaped by laws, policies, and conservation programs that emphasize instrumental values, such as the benefits wildlife provides to people, or intrinsic values, which recognize wildlife as having worth in its own right (Woolaston et al. 2021). But these frameworks do not always fully explain how people make everyday decisions in shared landscapes. People may also act toward wildlife based on relational values, choosing particular actions because of how they sustain relationships with other people, with different species, and with the moral or spiritual worlds to which they belong (Chan et al. 2016).

If relational values shape human–nature relationships, then societies that uphold relational moral philosophies provide an ideal context for investigating this influence. In Southern Africa, Sotho-Tswana societies uphold Botho, the ethic of personhood through relationships. Sotho-Tswana refers to a cluster of Bantu-speaking peoples located primarily in Southern Africa, specifically within Botswana, Lesotho, and parts of South Africa (Mphetolang 2009). This cultural and linguistic grouping explicitly includes the Basotho, Batswana, and Bapedi peoples, who speak Sesotho, Setswana, and Sepedi, respectively (Mokolatsie 2024). A Sotho-Tswana person is defined by Botho, a relational ethic which posits that authentic humanity is achieved only through interdependence, where the individual’s Seriti (dignity/vital force) is mutually constructed by acclaiming the dignity of others (Mphetolang 2009). This moral ontology requires the active practice of Boutlwelo botlhoko (compassion) and Tlhompho (respect) to maintain social harmony and extends moral obligations to the environment through totemism and to the economic realm through systems of reciprocal aid like mafisa (Mphetolang 2009). In this worldview, relationships with animals are embedded in and mediated by relationships among people (Sesiro 2023).

The Botho relational structure has implications for conservation. Within a Botho-informed worldview, people relate to animals through trusted human intermediaries (Kgari-Masondo 2014). It is customary, for example, to approach livestock only through the herder or owner, who alone can judge the animal's temperament and the safety of interaction (Pitikoe and Morojele 2017; Ndobochani 2022). Encounters with wildlife follow the same relational logic: people defer to elders, hunters, trackers, or other knowledgeable custodians who interpret animal behavior, assess risk, and guide appropriate responses (Thakadu 1997). Even animals with symbolic or spiritual significance, such as snakes or certain birds, are engaged through specialists or elders who mediate their meaning and relational implications (Denbow and Thebe 2006; Futhwa 2011). In Botho practicing communities, relationships with wild animals are not dyadic but triadic, constituted through people's relationships with intermediaries (Futhwa 2011; Sesiro 2023). Among Sotho-Tswana people, conservation authorities, rangers, and wildlife officers are intermediaries. When they fail to uphold what local residents understand as fairness, dignity, and mutual recognition, the entire relational system between humans institutions, and wildlife becomes destabilized (Sesiro 2023; LaRocco and Mogende 2024).

Pre-colonial indigenous communal governance and resource management in Botswana was shaped by Botho-informed practices; the ethic of humanness, interdependence, and mutual obligation guided local institutional behavior. Traditional institutions such as the chieftainship and the kgotla (village assembly) functioned as regulatory bodies, where chiefs held wildlife resources in trust to ensure sustainable utilization for the community (Mbaiwa and Darkoh 1997). Traditional leaders enforced unwritten norms regarding hunting seasons and selection criteria, such as restricting the killing of breeding animals and protecting rare species like the leopard for ceremonial use under specific instructions (Mbaiwa and Darkoh 1997). The kgotla served as a central institution for organizing communal hunts (letsholo), ensuring that the resulting meat and byproducts were distributed according to established social rules (Thakadu 1997). In the kgotla, guidance was shaped by the principle of Botho, which valued humanness and mutual respect; decisions were made together through open discussion and consensus, aiming to dignify everyone involved and maintain good relations among them (Kololo and Kari 2021). Consequently, decision-making within these institutions was adaptive and discursive, relying on debate and specific linguistic strategies to navigate social hierarchies and lived realities (Jacobsen 2017; Kololo and Kari 2021). Additionally, the cultural practice of totemism established a spiritual relationship between humans and nature, acting as a regulatory mechanism where specific tribes preserved specific species to maintain environmental stability (Chibvongodze 2016).

A distinguishing feature of these Botho-informed practices is that people are free to approach human intermediaries, elders, herders, healers, or other knowledgeable custodians to seek guidance about their relationships with animals such as snakes, cattle, or other wildlife (Futhwa 2011). Evidence indicates that intermediaries, ranging from herding peers to traditional chiefs, provide guidance that is deeply rooted in local contexts and essential for community survival. For example, Basotho herders rely on “local science” shared through “mutual communal interdependence” to address specific environmental challenges, ensuring advice is responsive to their immediate needs (Pitikoe and Morojele 2017). By contrast, government and conservation institutions rarely assume this mediating role in ways that align with local expectations (Noga et al. 2018). Rather than responding to community consultations or tailoring guidance to people's circumstances, conservation authorities typically define goals, management strategies, and acceptable behaviors based on scientific frameworks and institutional priorities (LaRocco and Mogende 2024). This creates a fundamental mismatch: while Botho-based relational systems rely on responsive, trusted human mediation, conservation governance often operates through top-down, expert-driven models that leave little room for relational negotiation (LaRocco and Mogende 2024). When conservation actors fail to act as accessible and culturally legitimate intermediaries, the relational pathway between people and wildlife becomes strained, contributing to conflict and eroding the conditions necessary for coexistence.

In Southern Africa, many conservation programs prioritize protecting large carnivores (Pooley et al. 2017; Somerville 2019). While these efforts have achieved some successes, human–carnivore conflict remains a persistent challenge, partly because of underlying human–human tensions between conservation actors and local livestock farmers (Brooks et al. 2010; Blackie et al. 2024). In Botswana, following the advent of colonial administration and continuing after independence, conservation has been led by the state and external actors (Mbaiwa and Darkoh 1997), displacing traditional governance and shaping local perceptions that responsibility for wildlife management now lies with outsiders rather than communities themselves (Nijhawan 2008; Jacobsen 2017). In some cases, these external actors, including government agencies, have sidelined and even supplanted local residents’ relationships with wildlife (Dawson et al. 2021; Naranjo et al. 2024). This dynamic deepens rifts when outsiders expect local people to demonstrate “tolerance” toward carnivores such as lions, even though external wildlife authorities have claimed responsibility for these animals, controlling interactions and benefits. Many residents believe that if outsiders manage and profit from wildlife, they should also take full responsibility for any damage to private property. Local cattle farmers often believe the government should bear responsibility for confining carnivores within national parks and game reserves. People do not expect to absorb the risks of living with wildlife when they have little influence over management decisions or benefit distribution. Research shows that, although most residents in Botswana absorb the risks of living alongside wildlife, they do so reluctantly and passively. These tensions highlight the need for a relational perspective on coexistence, one that examines not only interactions between humans and large carnivores but also the relationships between local communities and external conservation agents, including governmental and non-governmental organizations.

At first glance, it may seem that people in Botswana's rural communities are intolerant of lions. However, this perceived intolerance may, in reality, reflect dissatisfaction with the terms and structures of coexistence, rather than with the animals themselves (Serenari 2024). Residents may resent being excluded from decision-making or feel that the costs of living with carnivores, including livestock losses, human injury, or restrictions on land use, are unfairly distributed (Braczkowski et al. 2023). Again, much of what is framed as human–wildlife conflict may be better understood as “human–human conflict” (Madden 2004). When coexistence is forced upon people as a matter of law, it denies or ignores local human decisions and cultural norms. For coexistence to work, humans who live with carnivores need to be involved and their views and practices taken seriously. Understanding and practicing Botho is one way of doing so, and perhaps a critical dimension of participatory or community-oriented conservation in Botswana and beyond.

Findings from this study provide an empirical description of how Botho principles influence relationships among cattle farmers living with lions, between cattle farmers and lions, and between cattle farmers and conservation agents in the Ghanzi Wildlife Management Areas (GhWMAs) in the western Kalahari region of Botswana, a country in Southern Africa.

## What is Botho?

Botho is the word that labels what Sotho-Tswana people regard as the ideal way of being and relating to other living beings, and also with the non-living environment (Mokolatsie 2024). Having Botho means having a humble outlook on life (Le Grange 2015). Such humility is rooted in knowing that one is not entirely self-sufficient (Otlogetswe 2015). This knowledge enables individuals to behave in a manner that respects the needs of others for their well-being (Ewuoso and Hall 2019). Having Botho means that a person must acknowledge their own and others' capacity to affect each other for good or bad (Mokolatsie 2024). Botho acknowledges the natural, intrinsic worth of every being and bestows upon that individual the responsibility to restrain their negative capabilities and promote their positive capabilities for the benefit of others.

Closely related to Botho is the concept of etho, which refers to the cultivated character and ethical disposition of a person (Otlogetswe 2015). Setho is developed through cultural education, upbringing, and the moral instruction embedded in communal life (Mokolatsie 2024). As such, to be called a motho (person), in the moral sense, implies that one has undergone a process of ethical formation consistent with the values of one's culture. In Sotho-Tswana worldviews, this process entails internalizing virtues like respect, compassion, integrity, and accountability. The degree to which one manifests setho determines whether one can be said to truly possess Botho (Mokolatsie 2024).

Honoring the contribution of others to individual well-being requires receptivity to their perspectives, a disposition evidenced by the practice of listening (Jowah 2015). Listening also indicates that a person is aware of another's capacity and right to have independent thoughts (Sesiro 2023). Listening to others acknowledges the potential to benefit from their input, to show that their being matters, and to withhold negative words and express positive ones (Mokolatsie 2024).

Many Batswana conservationists show Botho by suspending assumptions and listening deeply to people in rural communities (Beaudette 2024). Such a practice, Beaudette (2024) argued, can improve a researcher's awareness of local knowledge and local capacity to co-produce solutions to complex challenges. This can be as simple as acknowledging the existence of others and the importance of connection for wellbeing and not isolating oneself. It manifests as simply greeting other people or accepting the presence of other things or beings and their right to be there.

The Setswana folktale of Ntiti and Ntitietsana (Johnson et al. 1987) is one example that teaches children the importance of Botho, or good character and respect. It is about two young princesses who, one day, went to the woods to gather firewood with other girls from the village. The older princess, Ntiti, was well-behaved and kind, while the younger princess, Ntitietsana, was rude and unkind. Ntitietsana knocked one of her toes on a small rock. She then insulted the rock. On their way back home, they found the rock had turned into a big hill and it would not let them pass. The only way to pass was to sing to the rock a song that one of the girls, the daughter of a traditional healer, had composed. But when it was Ntitietsana's turn, she sang rudely, so the rock would not let her pass. The two sisters were left behind and eventually got lost while trying to find an alternative route home. The girls came across an old lady who eventually helped them get on their way home. When sending off, the old lady gave the girls three stones to throw on the ground whenever man-eating giants pursued them. While running home, the girls used the rocks to create a forest that blocked the giants' way at one point and a river that swallowed the giants up at another point. A bird also helped the girls.

The folktale of Ntiti and Ntitietsana is a story that encodes the ethics of Botho in human relationships and in how people relate to the natural world. This story is found in primary school reading series, Molatedi wa Boraro. The story teaches that the land and its beings are relational partners, not inert objects to be used or insulted. In the context of conservation, the moral here is that coexistence requires not just strategy but character as well. From the perspective of Botho, this story suggests that positive relational outcomes can result when conservationists and cattle farmers alike approach each other, and the carnivores they share the land with, with humility, mutual respect, and a willingness to listen. As in the folktale, pathways to coexistence are blocked or opened not only by technical knowledge or outside interventions, but by how people choose to behave in those relationships.

## Study site and methods

The study is situated within the communal farming areas of the Ghanzi District, a region ecologically defined by the “Ghanzi Ridge”. Described as an “oasis in the center of the Kalahari,” this limestone outcropping is characterized by nutrient-rich soils that support “sweet grasses” and contains extensive underground aquifers allowing for sustainable wells. Historically, this favorable hydrology and vegetation attracted diverse pastoralist groups, including the Tjiherero, Bakgalagari, and Batswana, who established cattle posts in the area long before the colonial demarcation of the commercial “Ghanzi Farms” block. Consequently, the communal farming context here operates within a multi-ethnic landscape where livestock management remains deeply tied to the historical struggle for access to these critical groundwater sources. The focus of this paper is not on the private commercial ranches, but rather on communal grazing areas inside Wildlife Management Areas (WMAs) where cattle farming is done by multi-ethnic people.

Water resources in the communal farming area are largely managed through “borehole syndicates,” a form of private group ownership that emerged in the 1930s to secure reliable groundwater for livestock. Originally encouraged by the colonial administration to shift the financial burden of maintenance to users, these syndicates consist of groups of cattle owners who collectively fund the drilling, fuel, and repair of the pump. While organized around a managing committee to handle financial assessments, these groups often function as social entities, blending modern economic enterprise with traditional forms of community association. Crucially, while the syndicate technically owns only the water source, this exclusive access grants the group de facto control over the surrounding communal grazing land, effectively regulating range usage.

The Ghanzi farmlands in western Botswana are a prime example of semi-arid rangeland where humans, domestic livestock and wildlife live side by side with varying degrees of success. Though lions kill cows, horses, goats, and other livestock animals, and humans kill and harass lions, there are examples of coexistence. Livestock owners do not always respond with lethal removal when carnivores have preyed on their livestock. Only animals that become habituated to feeding on domestic animals are removed by translocation by the government.

The WMAs are designated by the government as multiple use zones, which include cattle posts, villages, and community zones with boreholes for livestock. The WMAs are also where lions are free roaming. Protecting habitat for wildlife is a primary function of the WMAs, but other activities like maintaining livestock are allowed (Parry and Campbell 1990; Twyman 2001). WMAs are also buffer zones between protected areas and human settlements (Jones 2008). Our study site is in the WMAs of GH10 and GH11, between the Central Kalahari Game Reserve (CKGR) and the Kalahari Trans frontier Park (KTP).

We used ethnographic methods of participant observation, field notes, and structured and open-ended interviews to look for relations of Botho between four main groups: Borehole syndicate members, Cheetah Conservation Botswana (CCB), a Botswana-based NGO, wildlife researchers affiliated with CCB and other conservation organizations, and the government Department of Wildlife and National Parks (DWNP). The first author led 12 months of research observing and participating in interactions between cattle farmers, DWNB and CCB conservationists, and lions, with a particular focus on the relationships and conversations between farmers and conservationists in consultation meetings, workshops, and conservation project activities (i.e., setting camera traps).

We accompanied CCB Field Officers, who are Batswana, and their collaborative wildlife researchers, who originate from Europe and the USA. We also interacted with cattle farmers in the syndicates, and people living in villages who are engaged in CCB projects, as well as those who are not cattle farmers. Additionally, we spoke with government extension officers in the towns and village chiefs. We had the opportunity to get to know and interact with many members of the community organizations and had many interactions with DWNP officers in meetings and workshops.

Through these ethnographic research, we made note of recurring practices that the participants used to interact with each other and when and how they showed Botho.

## Findings: How Botho enables coexistence

Over 12 months of observation and conversations with people about incidents involving lions in rural communities of the Kalahari, we saw government wildlife officers, conservationists, and farmers showing many customary practices that reflect the ethic of Botho.

Officers from the local division of DWNP know cattle farmers and villagers by name, knowing more about them than just how many lions or other predators they have reported to the DWNP. For example, during a workshop with farmers, a DWNP officer repeatedly addressed local farmers by name, referring to stories that reflected a history of collaboration over decades. When there were cases of depredation, the DWNP officers attending the case often worked with local trackers, some of whom were farm caretakers, to locate the lion or other animal they are seeking.

Batswana field officers working for conservation NGOs like CCB also practiced similar customs. For example, the CCB officers knew livestock owners and farm caretakers by name. Sometimes, the CCB staff passed near cattle farms when they were on their camera-trap checking routes. The farmers expressed appreciation when the officers stopped at their farm to greet and let them know they were in the area. The practice of greeting is valued because it is a form of recognition and acknowledgment of one another, which is fundamental to Botho. In Tswana communities, it is customary to announce one's presence when entering an area, a practice rooted in the communal tenure of land historically overseen by chiefs (Mbaiwa and Darkoh 1997). In today's context, this gesture remains a vital relational practice. It is not simply about safety, though it invites oversight and support in times of need It also signals relationship and humility. It reflects an acknowledgment of the social and moral fabric of the land, a recognition that one is not wholly self-sufficient, and a willingness to respect existing uses and values, even when legal rights may permit otherwise.

During the visits that government field wildlife officers made to the farms, the officers shared details about their work, the experiences of other farmers, and planned projects. The farmers and officers also discussed other non-work-related matters. For instance, an elderly female farmer shared how she supports her daughter, who is in tertiary school, using the proceeds from her farm. Field officers commonly switched to the local language, Sekgalagadi, to talk with locals. They greeted the farmers in town when they saw them, just to touch base. Thus, the officers and farmers focused not only on wildlife conservation activities, or “what the lions were doing,” but also on each other and in cultivating and nurturing human relationships.

During a workshop, a DWNP officer discussed the control of domestic livestock movement into the protected area. During this discussion, the officer referred openly to “*setho*.” The DWNP Officer presenting said, “*The law says that when a cow enters the CKGR, the DWNP officer must impound it…*” To this, a farm livestock caretaker in the audience immediately responded, “*Then we must kill the lion too!*” This exchange illustrated the conflict and hostility between cattle farmers and conservation agents over how to manage or cope with lions. However, government representatives, conservationists, and researchers draw on Botho to overcome these tensions and develop relationships that reflect empathy, reciprocity, and mutual respect. Such relationships are maintained through the exchange of gifts, periodic check-ins, familiarity, and inviting one another to participate in routine tasks. Positive relationships between individuals from different entities with different mandates emerge from these kinds of interactions that embody the ethic of Botho. Botho recognizes that one's humanity is linked to the humanity of every person in the world. It also recognizes that every person has the right to be treated with dignity. The wildlife management law may not allow for this empathy, but officers still use their discretion and apply Botho principles to their work. This was demonstrated when the DWNP officer explained, “*Even as we speak about the law as it currently stands, we have never acted like that. We usually just drive the livestock back. We haven't killed them. But the law has been written. That is something we need to be aware of. If I were to say, ”I have killed your cow in the protected area,“ you wouldn't be able to sue me. It is actually an illegal offense that we return your livestock from within the boundary of the park without killing them. If my superiors were to ask me why I didn't kill them, I have no legal protection. I could lose my job.*” It emerges from this account that despite the rigidity of formal conservation laws, officers navigate their roles with a sense of moral responsibility shaped by Botho. The rigidity of formal conservation laws does not yield to the context. For example, there is no physical boundary on the periphery of the protected area, and so wildlife and livestock do not recognize that boundary. By choosing empathy over enforcement, officers risk professional consequences to uphold contextual fairness and preserve relationships with the farmers.

In the instances where conservation NGOs, government agencies, and cattle farmers have cultivated good working relationships, there appears to be mutual empathy, reciprocity, and respect. There appears to be Botho. Farmers understand that government officials are human beings like themselves, and they are upholding the government's mandate when on duty. It is similar for the conservation agents, too. The officer mentioned above illustrated this, “*It is setho when the herder puts down his weapons before crossing into the park boundary to drive cattle back—that he enters unarmed. Then you wouldn't appear like you have intentions of poaching. I can't officially say ”yes” (that it's allowed), but I do understand.”* The word that the officer used, “setho,” is closely related to Botho. It refers to the cultivated character and moral disposition of a person (Otlogetswe 2015; Mokolatsie 2024). Setho is instilled through cultural education, upbringing, and the moral instruction embedded in communal life (Mokolatsie 2024). The degree to which one manifests setho determines whether one can be said to truly possess Botho (Mokolatsie 2024). Therefore, this is one example where we saw the conservation agent openly appealing to the farmers to draw on the cultural values of Botho when taking actions relating to livestock, wildlife and formal conservation laws.

Similarly, when livestock depredation occurs, the first external responders are the DWNP, followed by CCB and wildlife biologists. When a carnivore preys on a horse, cow, or goat of a farmer or threaten their safety, the farmer can be triggered to respond by killing the animal, which might be a lion, leopard, or wild dog. The law also permits farmers to do so. However, where relationships are good, the farmers use empathy for the conservationists and do all they can to avoid that removal. The farmers understand that the lethal removal of a species of global conservation concern goes against the interests of government wildlife officers and conservation NGOs. One farmer expressed that, “*Then in those cases you cannot kill the animal because its guardians are people whom we know. We are the same people, and we don't want to make their life difficult.*”

When cattle farmers, government wildlife officers, conservationists, and researchers disagree, it can appear as though carnivore conservation and cattle production are incompatible. Conflicts can occur when conservation agents focus on “educating,” “enforcing,” and “incentivizing” without building relationships with human residents, and/or without honoring existing human–wildlife relationships. In those cases, the farmers can strengthen solidarity between humans, through Botho, even while developing a resentment for the local wildlife. When Botho is no longer extended to the wildlife, there can be adverse outcomes for coexistence.

In Botswana, we have observed instances where residents of local communities seem to reinforce intra-human solidarity (Botho) in opposition to both conservation agents and wildlife. Animals are reclassified as outsiders, property of the state or as assets for the tourism industry. Local people view large carnivores as property of the state or external economic assets rather than community resources (Nijhawan 2008). Jacobsen observed that in Shorobe, a village excluded from wildlife tourism benefits, residents expressed alienation, explicitly asking why they should like animals from which they “get nothing,” implying these animals belong to the safari industry (Jacobsen 2017). This perception is reinforced by the legal framework where communities must rely on the Department of Wildlife and National Parks (DWNP) to manage conflict; for instance, after a fatal lion attack, it was government officials who were called to locate and kill the animal (Jacobsen 2017). Consequently, even where Botho remains central in shaping relationships among humans, it does not necessarily produce positive outcomes for coexistence. Botho upholds the idea that “the survival and harmony of the human community is the ground for morally right action” (Ewuoso and Hall's (2019). This suggests that ethical decisions are evaluated primarily based on their consequences for human communal life. Accordingly, Botho may legitimize actions that prioritize human well-being over the lives or interests of wild animals, particularly when wildlife poses threats to subsistence, safety, or social cohesion. In such contexts, Botho may be invoked to justify lethal or exclusionary responses toward carnivores or other wildlife perceived as dangerous.

Though human–human conflict can be the root of human–wildlife conflict, good relations between humans–wildlife officers, cattle farmers, and conservationists can be the root of coexistence. Botho can enable such relations between humans, which are essential for building good relations with large carnivores. In this light, at least in Southern Africa, Botho may be an effective tool for cultivating and nurturing coexistence.

## Conclusion: Breakdown of Botho between humans and lions

In this study, we asked how the African ethic of Botho—“I am because we are”—is one factor outside of laws, policies, and conservation programs that influences local agency, and why people choose to coexist with carnivores. Ethnographic observations and notes revealed that Botho operates strongly between people, particularly between wildlife officers and local cattle farmers in the Kalahari region of Botswana. The expression of Botho between social actors eased tensions, or human–human conflict, over wildlife. The impact on wildlife was that when people treated each other with Botho, they developed empathy for one another, which sometimes extended to the wildlife. That is to say, local residents treated the wildlife kindly because the social actors tasked with wildlife conservation showed connection, humility, and empathy. In the Kalahari, the state and international and conservation organizations tend to mediate human relationships with wildlife. It is as if cattle farmers relate to wildlife through government wildlife officers, researchers, and NGOs. For these reasons, Botho may be essential for turning conflict with wildlife into coexistence, building on and respecting the choices humans are already making to “tolerate” and live with carnivores. Botho can also cultivate empathy and understanding between different social actors, which can benefit wildlife. This is a powerful if indirect way Botho is a path to coexistence.
